# Annotations

**Published:** 1897-02-20

**Authors:** 


### Annotations.

### The Death Tax upon Poverty.

A study of the report of the Medical Officer of Health to the London County Council for 1895, which has just been issued, shows, among other things, the extraordinary difference in the death-rates in the various parts of the metropolis. Hampstead rejoices in a death-rate of only 14·5 per 1,000, while the inhabitants of the parish of St. Luke died at the rate of 31·9 per 1,000 in the year; in fact, there are three parishes in which people die at more than twice the rate they do in Hampstead. Does this mean that the conditions of life are twice as good in Hampstead as in St. Luke's, St. George's-in-the-East, or in Limehouse? The problem is not so simple as might appear. These three districts all have high infantile death-rates, they all have extremely high rates for zymotic disease, people dying from these diseases, both in St. Luke's and in St. George's-in-the-East, at more than five times the rate which prevails in Hampstead. Again, all these three districts have abnormally high death-rates from phthisis, while Hampstead has an abnormally low one. Is it, then, true that to live in Hampstead means health, while to live in St. Luke's or in St. George's-in-the-East means such a shortened span of life as is expressed by these figures? Not at all. The constitution of the population must be considered besides their surroundings. The successful and the prosperous occupy the heights of Hampstead. The poor, the unsuccessful, the failures in life who have failed largely from want of constitutional vigour, drift into the slums of the East and South of London. Consumption is common, not entirely because slum surroundings produce it, but because consumption itself produces incapacity for work and drives the sufferer to the slums. Infectious fevers prevail because of the overcrowding that poverty entails; but, so far as fevers tell of sanitary conditions, it is to be noted that some of the poorest and most crowded central districts suffer but little from typhoid fever, some, in fact, far less than Hampstead itself. The lowest typhoid death-rate occurred in Holborn, where no deaths from that cause took place, and the two next lowest rates were in St. Saviour's, Southwark, and in St. Giles! Death-rates must not be too readily accepted as guides to sanitary condition. The poor, the miserable, and the constitutionally feeble flock together, and wherever they accumulate there the death-rate tends to run up.

### Soldiering and Health.

All the world seems to be charged with the feeling that England may in no long time be called upon to fight for her existence, not at sea only, but also by land, and within reach of the sound of Bow Bells. Some few are beginning to frown at the supposed necessity for turning the nation into a camp, and the whole male population into an army. To be frank, we do not agree with them. If the question of health be considered important, there are few things, if any, which would more immediately and more effectually induce the improvement of the national physique than a course of soldiering. It has come to be the case in this country that everybody above the level of the mere workman looks to the making of a fortune as the natural result of his activities. Not only so, but he expects to make it in a short time, and then either to enter upon a public career, or to retire into idleness and self-indulgence for the rest of his natural life. Now, if physiology teaches anything at all, she teaches that nature has no intention of this kind whatever for any considerable proportion of human beings. Nature's intentions seem to be to make us active and healthy; healthy because active, as long as we live; and when we can no longer be active, nature lets us droop and die. Soldiering would convert us all to activity, if it did nothing else. It would keep our young men out of offices and shops at a time of life when offices and shops are the very worst places in the world for them. It would develop arms and legs in due proportion to wits, and would make men less keen-witted and cunning, but more honest. From the medical point of view, the health part, that is, we shall not regret if the threatenings of our neighbours should enforce upon the nation the necessity of calling upon every young man to give a year or two of his life to soldiering.

### London and the Plague.

In view of the tendency which the plague has shown to spread to various parts of India, and the obvious alarm felt by many Continental governments in regard to the possibility of its being introduced into Europe, we have made inquiries as to what precautions are being taken in London to prevent its becoming implanted among us. Our first line of defence lies with the Port Sanitary Authorities, who inspect shipping as it arrives in the Thames. We understand, however, that a careful watch is also being kept on the lodging-houses which are most frequented by sailors, lascars, and others engaged in the East India trade, and it does not seem probable that the disease would spread far without being discovered. If cases arose they would have to be dealt with by the local sanitary authorities, and here is the weak spot. We doubt if a single sanitary authority in London has a properly equipped hospital for general isolation purposes, and as to the hospitals of the Asylums Board they would have no right to accept cases of plague, that not being a notifiable disease. Then as regards the provision of "shelters," *i.e.*, places in which families may be housed while their dwellings are being disinfected, the local authorities are sadly behindhand. Every effort is, however, being made to hasten their fulfilment of what is a duty laid by law upon them. Next, as to disinfection. Many of the local sanitary authorities of London are in a lamentable condition of unpreparedness, although much better off than they were two or three years ago; and, as for any completely organised arrangement for immediately cleansing an infected house, scrubbing it and liming it, and clearing out the drains, such as was used with great effect when cholera threatened Hull in 1893, we doubt if such an organisation has ever been dreamed of in many of the sanitary districts of the metropolis. Sanitary knowledge keeps far ahead of sanitary practice, which is not altogether creditable to London. We have every confidence in our power of putting a limit to any outbreak that may occur. Unfortunately, however, if plague were to be brought to London, there is every probability of many lives being lost before our full sanitary forces could be brought to bear on the disease.

